# Supplementary material for: RDP5: a computer program for analyzing recombination in, and removing signals of recombination from, nucleotide sequence datasets
Source: Virus Evol. 2020 Apr 12;7(1):veaa087. doi: 10.1093/ve/veaa087 (PMC8062008; doi:10.1093/ve/veaa087)
Supplement: veaa087_Supplementary_Data [file veaa087_supplementary_data.zip › RDP5.5.docx]

RDP5: A computer program for analysing recombination in, and removing signals of recombination from, nucleotide sequence datasets

Darren P Martin, ^1*^† Arvind Varsani^2,3^, Philippe Roumagnac^4^, Gerrit Botha^1^, Suresh Maslamoney^1^, Tiana Schwab^5^, Zena Kelz^1^, Venkatesh Kumar^1,6^ and Ben Murrell^6^

^1^Department of Integrative Biomedical Sciences, Computational Biology Group, Institute of Infectious Disease and Molecular Medicine, University of Cape Town, Anzio Road Observatory 7549, Cape Town, South Africa

^2^The Biodesign Center for Fundamental and Applied Microbiomics, Center for Evolution and Medicine, School of Life Sciences, Arizona State University, Tempe, AZ 85287-5001, USA

^3^Structural Biology Research Unit, Department of Integrative Biomedical Sciences, University of Cape Town, Observatory, Cape Town 7701, South Africa

^4^ BGPI, Univ Montpellier, CIRAD, INRAE, L’Institut Agro, Montpellier, France

^5^Institute of Bioengineering, École Polytechnique Fédérale de Lausanne, Lausanne CH-1015, Switzerland.

^6^ Department of Microbiology, Tumor and Cell Biology, Karolinska Institutet, Stockholm, Sweden

*Corresponding author: E-mail: darrenpatrickmartin@gmail.com

†Darren P. Martin: http://orcid.org/0000-0002-8785-0870

**Abstract**

For the past 20 years the recombination detection program (RDP) project has focused on the development of a fast, flexible and easy to use Windows-based recombination analysis tool. Whereas previous versions of this tool have relied on considerable user-mediated verification of detected recombination events, the latest iteration, RDP5, is automated enough that it can be integrated within analysis pipelines and run without any user input. The main innovation enabling this degree of automation is the implementation of statistical tests to identify recombination signals that could be attributable to evolutionary processes other than recombination. The additional analysis time required for these tests has been offset by algorithmic improvements throughout the program such that, relative to RDP4, RDP5 will still run up to five times faster and be capable of analysing alignments containing twice as many sequences (up to 5000) that are five times longer (up to 50 million sites). For users wanting to remove signals of recombination from their datasets before using them for downstream phylogenetics-based molecular evolution analyses, RDP5 can disassemble detected recombinant sequences into their constituent parts and output a variety of different recombination-free datasets in an array of different alignment formats. For users that are interested in exploring the recombination history of their datasets, all the manual verification, data management and data visualization components of RDP5 have been extensively updated to minimize the amount of time needed by users to individually verify and refine the program’s interpretation of each of the individual recombination events that it detects.

**Introduction**

Recombination and genome component reassortment are processes that strongly impact the evolution of many virus species. The patterns of nucleotide sequence variation created in virus genomic sequence datasets by these processes (which are hereafter collectively referred to as recombination) can seriously undermine the accuracy of phylogenetics-based molecular evolution analyses (Schierup and Hein 2000a,b; Scheffler, Martin, and Seoighe 2006; Arenas and Posada 2010). It is therefore frequently desirable to test nucleotide sequence datasets for evidence of recombination and, when such evidence is found, take steps to minimize the impacts of detected recombination events on downstream analyses of these datasets.

Of the many available computer programs for analysing recombination (see <http://bioinf.man.ac.uk/robertson/recombination/programs.shtml>; Martin, Lemey, and Posada 2011), recombination detection program (RDP) is presently one of the most commonly used. Successive versions of RDP have applied an expanding array of recombination event detection, recombination breakpoint demarcation, and recombinant sequence identification methods, all applied in unison, to yield detailed descriptions of how recombination may have impacted the evolution of any given set of aligned nucleotide sequences (Martin et al 2015). The accuracy of these descriptions, however, frequently depended on the amount of effort users were willing to put into exploring the many similarly plausible ways in which the detected patterns of recombination may have arisen. A guiding principle during the development of RDP5, the latest version of the RDP series, has therefore been a minimization of the amount of time that users need to invest in detecting and removing signals of recombination from nucleotide sequence datasets.

**Generation of recombination-free datasets**

RDP5 can take as input an aligned nucleotide sequence dataset, automatically identify and characterize individual recombination events that are evident within that dataset, and output modifications of the input alignment within which all signals of detectable recombination have been removed. The different types of modified recombination-free multiple sequence datasets that RDP5 can output include alignments where: (1) recombinant sequences have been removed; (2) fragments of sequence derived through recombination have been removed; (3), recombinant sequences are split up into their constituent parts; and (4) the input alignment is divided into multiple different gene/genome sub-region alignments based on the locations of detected recombination breakpoints (Supplementary Figure 1).

**Query vs reference scans for recombination**

In addition to the fully exploratory recombination analysis modes found in previous versions of the program (Martin et al., 2015), RDP5 also includes a new highly automated “query vs reference” analysis mode such as that found in the programs REGA (de Oliveira et al. 2005) and jpHMM (Schultz et al. 2006). Unlike with the default fully exploratory recombination analysis mode where every sequence is tested for evidence of recombination, the query vs reference mode will test a user-defined set of query sequences for evidence that they originated through recombination between a user-defined set of reference sequences. Such an analysis mode is well suited to analysing patterns of recombination between two or more groups of viruses that have only recently had the opportunity to start recombining with one another: such as in an individual patient that has been infected with two distinct variants of a virus (Sheward et al., 2018), or within a geographical region where multiple distinct genetic variants of a virus have recently started co-circulating.

To minimize the amount of effort needed to define reference sequences and/or groups of reference sequences, RDP5 will automatically identify sequences as queries and references based on simple sequence naming rules (see the manual provided with RDP5 for these rules). These naming rules can also be used to group reference sequences into different reference types.

**Automated sequence annotation**

Given an accessible internet connection, RDP5 will automatically annotate the genomic features of input virus sequence datasets using the curated NCBI virus reference sequence database (<https://www.ncbi.nlm.nih.gov/genome/viruses/>). Annotation is useful in the context of generating recombination-free datasets because it enables RDP5 to output gene sequence alignments that are suitable for downstream codon-focused analyses of natural selection. The gene sequence alignments that RDP5 produces can account for variation in the positions of gene start and stop codons, intron splicing (wherever intron donor and acceptor sites are annotated in the NCBI reference sequence records), portions of genes that are expressed in two or more different open reading frames (these regions can be excluded) and real and/or artefactual frame-shift mutations (partial codons can be removed).

Sequence annotation is also useful for testing the selective processes that impact patterns of recombination detected within sequence datasets. Given a set of annotated genome sequences recombination breakpoint distribution tests in RDP5 will now automatically detect whether observed recombination breakpoint distributions vary between: (1) coding and non-coding regions; (2) different genes; and (3) the edges and internal regions of genes.

**Detection of potential false-positive recombination signals**

A crucial facilitator of the highly automated recombination analyses that RDP5 can perform is the inclusion of tests that are specifically designed to detect and flag potential false-positive signals of recombination. Besides automatically testing whether each of the recombination signals that RDP5 detects might be attributable to sequence misalignment (which is a major contributor to false positive signals of recombination), the program will also detect and flag as suspicious any detectable recombination signals that may have arisen through evolutionary processes other than recombination. Specifically, RDP5 uses the PHI test (Bruin, Philippe, and Bryan, 2006) and 4-gamete test (McVean, Awadalla, and Fearnhead 2002) adapted versions of the homoplasy test (Maynard Smith and Smith 1998) to flag apparent recombination signals that are potentially attributable to a combination of inter-lineage and inter-site mutation-rate variation rather than recombination (Bertrand, Johansson, and Norberg, 2016).

**RDP5CL: a command-line version of RDP5**

For instances where users would like to integrate RDP5 into an analysis pipeline, a separate command-line driven version of the program, RDP5CL, is distributed with RDP5. RDP5CL will take as input a multiple sequence alignment in any standard alignment file format and, contingent on command line switches, output any of various different types of recombination-free alignments, recombination breakpoint distribution plots and/or maximum likelihood (Stamatakis 2014) phylogenetic trees accounting for recombination.

**Improved computational performance**

Despite the additional tests that RDP5 carries out during the characterization of detected recombination events, it is still able to analyse any given dataset between two and five times faster than RDP4 could (Supplementary Table 1). To achieve this, RDP5 implements a multitude of algorithmic improvements such as multi-core CPU-level parallelization and intensive use of lookup tables for bootstrap, permutation, likelihood and probability calculations. Additionally, while previous versions of RDP were restricted to using 2GB of available RAM (which is standard for 32-Bit Windows programs), RDP5 can utilize 4GB of RAM even under 32-bit Windows operating systems. This, together with improvements in memory management, means that RDP5 can analyse datasets that contain up to twice as many sequences that are each five times longer than those which RDP4 could manage.

The improved computational performance of RDP5 also extends to the manual recombination-signal verification, data management and data visualization components of the program. These enhancements will substantially decrease the amount of time that it takes users to manually verify and refine RDP5’s interpretation of each of the individual recombination events that it detects.

**Operational limits**

RDP5 will work on computers with Windows 7/Vista/8/10 operating systems and can also be installed to run under Windows 7 emulators running on computers with MacOSx and UNIX operating systems.

RDP5 can be used to productively analyse datasets containing up to 400 million nucleotides within 24 hours on a standard 8-core 2.5 GHz processor with >4 GB of RAM. Such datasets might, for example, consist of 120 3.0-Mb-long bacterial genome sequences, or 4000 10-kb-long viral genome sequences. With default program settings, RDP5 can analyse 100 10-kb-long sequences in under five minutes on a standard desktop computer.

**Availability**

RDP5 is available for free download from http://web.cbio.uct.ac.za/~darren/rdp.html. It is distributed with an extensive manual that contains (1) detailed descriptions of the various methods implemented in the program; (2) a step-by-step guide describing how to create and analyse datasets for recombination detection; (3) instructions on how to run completely automated analyses from the command-line using RDP5CL; and (4) information on how to run RDP5 on Mac and Linux computers.

**Acknowedgements**

ZK was funded by the South African National Research Foundation. BM was supported by the Swedish Research Council (grant no. 2018-02381). DPM was supported by the H3Africa

**References**

Arenas, M., and Posada, D. (2010) ‘The Effect of Recombination on the Reconstruction of Ancestral Sequences’, Genetics, 184:1133–9.

Bertrand, Y. J. K., Johansson, M., Norberg, P. (2016) ‘Revisiting Recombination Signal in the Tick-Borne Encephalitis Virus: A Simulation Approach’, PLoS One, 11: e0164435.

Bruin, T. C., Philippe, H., Bryant, D. (2006) ‘A simple and robust statistical test for detecting the presence of recombination’, Genetics, 172: 2665–81.

de Oliveira, T., Deforche, K., Cassol, S., Salminen, M., Paraskevis, D., Seebregts, C., Snoeck, J., Janse van Rensburg, E., Wensing, A. M. J., van de Vijver, D. A., Boucher, C. A., Camacho, R., Vandamme, A-M. (2005) ‘An Automated Genotyping System for Analysis of HIV-1 and Other Microbial Sequences’, Bioinformatics, 21: 3797–800.

Martin, D. P., Murrell, B., Golden, M., Khoosal, A., Muhire, B. (2015) ‘RDP4: Detection and analysis of recombination patterns in virus genomes’, Virus Evolution:1:vev003.

Maynard Smith, J., Smith, N. H. (1998) ‘Detecting Recombination from Gene Trees’, Molecular Biology and Evolution, 15:590-9.

McVean, G., Awadalla, P., Fearnhead, P. (2002) ‘A Coalescent-Based Method for Detecting and Estimating Recombination from Gene Sequences’, Genetics 160:1231–41.

Scheffler, K., Martin, D. P., and Seoighe, C. (2006) ‘Robust Inference of Positive Selection from Recombining Coding Sequences’, Bioinformatics, 22: 2493–9.

Schierup, M. H., and Hein, J. (2000a) ‘Consequences of Recombination on Traditional Phylogenetic Analysis’, Genetics, 156: 879–91.

Schierup, M. H., and Hein, J. (2000b) ‘Recombination and the Molecular Clock’, Molecular Biology and Evolution, 17: 1578–9.

Schultz, A.-K., Zhang, M., Leitner, T., Kuiken, C., Korber, B., Morgenstern, B., Stanke, M. (2006). ‘A Jumping Profile Hidden Markov Model and Applications to Recombination Sites in HIV and HCV Genomes’, BMC Bioinformatics, 7:265.

Sheward, D. J., Marais, J., Bekker, V., Murrell, B., Eren, K., Bhiman, J. N., Nonyane, M., Garrett, N., Woodman, Z.L., Abdool Karim, Q., Abdool Karim, S. S., Morris, L., Moore, P.L., Williamson, C. (2018) ‘HIV Superinfection Drives De Novo Antibody Responses and Not Neutralization Breadth’, Cell Host Microbe, 24:593-9.

Stamatakis, A. (2014) ‘RAxML Version 8: A Tool for Phylogenetic Analysis and Post-Analysis of Large Phylogenies’, Bioinformatics, 30: 1312–3.

**Tables**

Supplementary Table 1. Run times (in seconds on a 3.1 GHz Dell XPS7 with 8 cores) of RDP5, RDP4.101 (the most up-to-date version of RDP4) and RDP4.46 (the version of RDP4 published in the last RDP paper) with default settings on the four example datasets that are provided with the RDP5 installation. The main difference between RDP4.101 and RDP4.46 is that RDP4.101 contains ported versions of the false positive checks that were developed for RDP5: i.e the slower run times of RDP4.101 relative to RDP4.46 reflect the computational costs of these additional tests.

| Dataset | # Taxa | # Sites | RDP4.46 | RDP4.101 | RDP5 |
| --- | --- | --- | --- | --- | --- |
| Example2(A-J-cons-kal153).fas | 8 | 9954 | 4.4 | 8.2 | 1.9 |
| Example1(PotySeqs).fas | 25 | 9594 | 30.7 | 40.2 | 13.9 |
| Example3(FMDV).rdp | 91 | 8294 | 665 | 907 | 231 |
| HIV Example.rdp | 274 | 9557 | 20745 | 15864 | 3662 |

**Figure Legend**

Supplementary Figure 1. Various ways in which RDP5 can be used to remove evidence of recombination from nucleotide sequence datasets. Starting with a five sequence alignment where one of the sequences is recombinant (sequence R), it is possible to use RDP5 to produce four different types of datasets that contain no obvious evidence of recombination. Each rectangle on the left represents a nucleotide sequence, the bold lines indicate portions of the dataset where nucleotide sequence characters have been replaced by gap or missing data characters, and colours of regions in the rectangles represent the relationships between the sequences. Sequence “R” has one parent resembling sequence 2 and one parent representing sequence 3. The dendrograms on the right represent phylogenetic trees that might be produced from these datasets once they have been processed to remove evidence of recombination.
