# Supplementary figures and images for: RDP5: a computer program for analyzing recombination in, and removing signals of recombination from, nucleotide sequence datasets
Source: Virus Evol. 2020 Apr 12;7(1):veaa087. doi: 10.1093/ve/veaa087 (PMC8062008; doi:10.1093/ve/veaa087)

## Slide 1
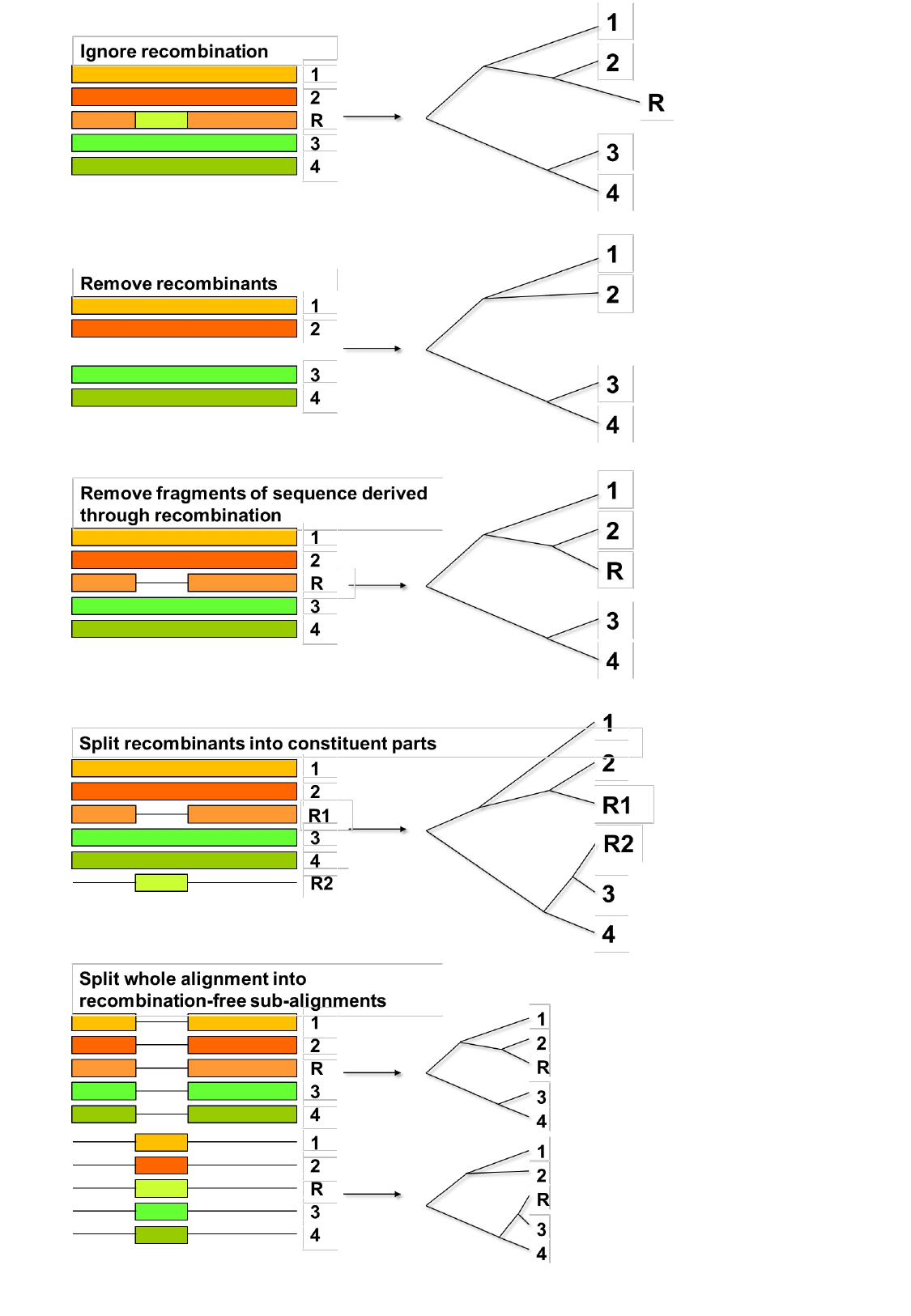

Supplement: veaa087_Supplementary_Data [file veaa087_supplementary_data.zip › Supplimentary Figure 1.pptx]
